# YC‐4‐3, a Novel Glycogen Synthase Kinase 3β Inhibitor, Alleviates the Endoplasmic Reticulum Stress of Macrophages in Primary Immune Thrombocytopenia

**DOI:** 10.1002/advs.202412515

**Published:** 2025-03-07

**Authors:** Pengcheng Xu, Lili Ji, Yanxia Zhan, Yang Ou, Xia Shao, Xibing Zhuang, Fanli Hua, Feng Li, Hao Chen, Yong Chu, Yunfeng Cheng

**Affiliations:** ^1^ Department of Hematology Zhongshan Hospital Fudan University Shanghai 200032 China; ^2^ Department of Hematology Zhongshan Hospital Qingpu Branch Fudan University Shanghai 200032 China; ^3^ Center for Tumor Diagnosis & Therapy Jinshan Hospital Fudan University Shanghai 201508 China; ^4^ Department of Thoracic Surgery Zhongshan – Xuhui Hospital Fudan University Shanghai 200031 China; ^5^ Department of Medicinal Chemistry School of Pharmacy Fudan University Shanghai 201203 China; ^6^ Institute of Clinical Science Zhongshan Hospital Fudan University Shanghai 200032 China

**Keywords:** endoplasmic reticulum (ER) stress, glycogen synthase kinase 3β (GSK‐3β), macrophages, none‐catalytic ATP‐binding site, primary immune thrombocytopenia

## Abstract

Primary immune thrombocytopenia (ITP) is a heterogeneous autoimmune disease, characterized by decreased platelet count and increased risk of hemorrhage, in which macrophages play an important role in the pathogenesis. This study aims to explore the effects of YC‐4‐3, the patented chemical synthesis of benzothiazepinone compounds (BTZs), a novel GSK‐3β inhibitor (GSK‐3βi), on macrophages in ITP. The expressions of GSK‐3β in monocytes are tested. The effects of GSK‐3βi (YC‐4‐3) on macrophages of ITP patients are examined and validated in passive and active murine models. Signal pathway enrichment analysis is performed. The interaction proteins of endoplasmic reticulum (ER) stress and GSK‐3β are explored. The GSK‐3β^+^ cells in monocytes are increased in newly diagnosed ITP patients and decreased in treatment‐response patients. YC‐4‐3 can restrain the proinflammatory differentiation, phagocytosis, and cytokine generation of macrophages and alleviate thrombocytopenia in ITP. YC‐4‐3 suppresses the PI3K/mTOR/Akt, NFκB/IκBα, and MAPK pathways, as well as the ER stress signal pathway. YC‐4‐3 directly interacts with the protein chaperone Bip. YC‐4‐3, a patented GSK‐3βi, can modulate the inflammatory status of macrophages and improve the thrombocytopenia in ITP by directly interacting with ER stress response. YC‐4‐3 may be a novel potential therapeutic agent for ITP.

## Graphical abstract

A patented GSK‐3β inhibitor (GSK‐3βi), new benzothiazepinone compounds (BTZs), YC‐4‐3, can modulate the inflammatory status of macrophages in primary immune thrombocytopenia (ITP), and improve the thrombocytopenia in murine ITP models by directly interacting with endoplasmic reticulum (ER) stress response. YC‐4‐3, as a potential medicine for ITP, is a substantial evidence for a novel therapeutic strategy in ITP.

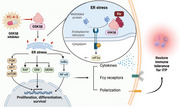

## 1 Introduction

Primary immune thrombocytopenia (ITP) is a heterogeneous autoimmune disease characterized by decreased platelet count and varying degrees of hemorrhage.^[^ ^1^, ^2^, ^3^, ^4^, ^5^ ^]^ The pathophysiology of ITP is complex and remains to be explored, which mainly includes pathogenic anti‐platelet autoantibodies and T cell‐mediated platelet destruction in the periphery and the impaired megakaryocyte function in the bone marrow.^[^ ^6^, ^7^, ^8^, ^9^, ^10^ ^]^ Among these factors, macrophages have been proved to play a major role in the pathogenesis of ITP, which can both directly mediate the destruction of platelets by Fcγ receptors and act as antigen‐presenting cells to stimulate immune responses.^[^ ^11^, ^12^ ^]^

Glucocorticoids and immunoglobulins, the first‐line treatments of ITP, and other therapeutic agents, such as thrombopoietin receptor agonists, spleen tyrosine kinase (Syk) inhibitors, and Bruton tyrosine kinase inhibitor (BTKi),^[^ ^13^, ^14^ ^]^ can play therapeutic roles by regulating Fcγ receptor‐mediated macrophage phagocytosis. Nevertheless, there are still some refractory ITP patients with poor response. Recently, anti‐CD38 targeted therapy boosted platelet levels by inhibiting antibody‐dependent cell‐mediated cytotoxicity, clearing plasma cells, and decreased the number of CD38+ monocytes and macrophages, especially macrophages, inhibiting the macrophages phagocytic system in the spleen of passive ITP mice models.^[^ ^15^ ^]^

Macrophages mainly differentiate into two phenotypes: pro‐inflammatory (M1) and anti‐inflammatory (M2) macrophages. M1 macrophages are characterized by high antigen presentation ability and expression of proinflammatory cytokines such as tumor necrosis factor α (TNFα), interleukin IL‐1β, and IL‐6, while M2 macrophages mainly secrete anti‐inflammatory factors, such as transforming growth factor β (TGF‐β) and IL‐10, and exert anti‐inflammation effects.^[^ ^16^, ^17^ ^]^ Previous studies found that in ITP patients, the number and activation of M1 macrophages are significantly higher than that of normal controls, correspondingly with impaired M2 macrophages, and treatments such as glucocorticoids could shift macrophages toward the M2 direction.^[^ ^18^, ^19^ ^]^ These results suggest that macrophages are indispensable in the pathogenesis of ITP, and restoring the differentiation of macrophages can be an important therapeutic direction for ITP.

Glycogen synthase kinase 3β (GSK‐3β) is a ubiquitously expressed serine/threonine kinase and has two ubiquitously expressed forms, GSK‐3α and GSK‐3β in mammals.^[^ ^20^ ^]^ It is known that GSK‐3β plays a central role in a variety of signaling pathways that are relevant to macrophage polarization, migration, and proliferation in some diseases, such as atherogenesis, ischemia, and obesity‐induced inflammation.^[^ ^20^, ^21^, ^22^, ^23^ ^]^ GSK‐3β inhibitors (GSK‐3βi) have been found to be therapeutic for several diseases such as pulmonary fibrosis, rheumatoid arthritis, as well as atherosclerosis by modulating the inflammatory state of macrophages and inhibiting the secretion of proinflammatory cytokines.^[^ ^20^, ^24^, ^25^ ^]^ However, few small molecule inhibitors could distinguish between GSK‐3β and its ubiquitously expressed form of GSK‐3α, limiting the clinical application of GSK‐3βi.^[^ ^20^ ^]^ Previously, we reported, Candidate 4‐3(named YC‐4‐3), a covalent benzothiazepinone compound (BTZs) as a novel GSK‐3βi.^[^ ^26^ ^]^ YC‐4‐3 showed much higher activity and selectivity in proteins and good efficacy and safety in leukemia cell lines and acute promyelocytic leukemia (APL) animals than traditional non‐covalent drugs.^[^ ^26^ ^]^ In a pilot study, YC‐4‐3 also showed immunoregulatory effect. Especially, YC‐4‐3 acts as an irreversible covalent mechanism with GSK‐3β, which might lead to more advantages than traditional non‐covalent drugs, such as higher specificity and stronger binding ability to target,^[^ ^27^ ^]^ longer residence time and better pharmacokinetic characteristics in vivo,^[^ ^28^ ^]^ and lower dose and higher therapeutic index leading to better safety in treatment.^[^ ^29^ ^]^ These characteristics suggested that YC‐4‐3 might be useful in the treatment of disease.

Endoplasmic reticulum (ER) stress maintains cellular protein homeostasis under endogenous or exogenous stimuli and affects various cell signaling processes. There are three key signal activators for ER stress, IRE1, PERK, and ATF6, that give rise to separate branches of the response.^[^ ^30^ ^]^ Studies found that ER stress can induce the activation of GSK‐3β, which is related to the PERK signaling pathway.^[^ ^23^, ^31^ ^]^ In macrophages, ER stress was found to regulate apoptosis, survival, polarization, and lipid accumulation in several conditions.^[^ ^23^, ^30^ ^]^ These findings imply a close relationship between GSK‐3β and ER stress in regulating macrophages, although the exact mechanism is still not clear.

This study aimed to evaluate the expression of GSK‐3β in monocytes in ITP patients, to examine the regulating effects of the novel GSK‐3βi YC‐4‐3 on macrophages in ITP, to explore the specific mechanisms of GSK‐3β, and to provide evidence for YC‐4‐3, this novel GSK‐3βi as a new potential therapeutic target for ITP, and to investigate its unique mechanism in the treatment of ITP.

## 2 Experimental Section

### 2.1 Patients

From January 2022 to October 2022, a total of 40 ITP patients according to the ITP diagnosis criteria proposed by the international working group^[^ ^1^ ^]^ and 26 healthy controls were enrolled. Detailed information of patients was shown in Table 1 (Supporting Information). The ITP patients received stranded high‐dose dexamethasone (HD‐DXM) therapy (40 mg/day × 4 consecutive days)^[^ ^1^ ^]^ and blood samples were collected by ten days after treatment. Patients with platelet counts ≥ 100 × 10^9^ /L and without any hemorrhage were defined as complete response (CR), patients with platelet counts ≥ 30 × 10^9^ /L and at least twice of the baseline were defined as partial response (PR), or else no response (NR).^[^ ^32^ ^]^ The study was approved by the Institutional Review Board of Zhongshan Hospital, Fudan University and conducted in accordance with the Declaration of Helsinki.

### 2.2 Materials and Animals

YC‐4‐3, an irreversible covalent GSK‐3βi, was synthesized and verified based on previous study.^[^ ^26^ ^]^ Wild type (WT) C57BL/6 mice (6–8 weeks of age, female) were purchased from Shanghai Jiesijie laboratory animal company (Shanghai, China). Severe combined immunodeficient (SCID) mice with a C57BL/6 background (J001913, 6–8 weeks of age, female) and C57BL/6 CD41 knockout mice were kindly provided by Drs. Jun Peng and Ming Hou (Qilu Hospital, Shandong University, Jinan, China). All mice were housed under pathogen‐free conditions with unrestricted access to food and water, with a 12‐h light‐dark cycle. The animal room was kept at a constant temperature of 23 °C, and 40% humidity. Animal studies were approved by the Animal Care and Use Committee of Zhongshan Hospital, Fudan University.

### 2.3 Murine ITP Models

For the passive ITP model, C57BL/6 mice were randomly divided into YC‐4‐3 group and control group. All mice received q.d. anti‐mouse CD41 monoclonal antibody (clone MWReg30; BD Biosciences, CA, USA) injection intraperitoneally at an initial dosage of 68 µg kg⁻^1^ for the first two days, followed by a dose increase of 34 µg k^−1^g each day until day 7.^[^ ^33^ ^]^ Mice in the GSK‐3βi group were administrated with YC‐4‐3 intraperitoneally at the dosage of 5mg/ kg/d from day 1 to day 7, while mice in the control group received the same volume of solvent placebo injection. Peripheral platelet counts of mice were monitored every other day. All mice were sacrificed on day 7, and single cell suspensions were prepared from peripheral blood, spleen, and bone marrow for flow cytometry or cell culture.

For the active ITP model,^[^ ^34^ ^]^ CD41 knockout mice were immunized with WT platelets weekly for 7 weeks. The serum immunoglobulin G (IgG) antiplatelet antibody in CD41 knockout mice was monitored by flow cytometry. Mice with antibody positive rate of more than 40% at 1:3200 titers were considered immunized successfully.^[^ ^35^ ^]^ Irradiated SCID mice were randomly divided into YC‐4‐3 group and control group. Each animal received transplantation of 5 × 10^4^ splenocytes from successfully immunized CD41 knockout mice to induce ITP. Mice in the GSK‐3βi group received intraperitoneal injection of YC‐4‐3 at the dosage of 5mg/ kg q.o.d. from the first day of splenocyte transplantation to week 5; Animals of the control group received the same volume of solvent placebo injections. Peripheral platelet counts of mice were monitored weekly. All mice were sacrificed at week 5, and single cell suspensions were prepared from peripheral blood, spleen, and bone marrow for flow cytometry.

### 2.4 Cell Culture

Peripheral blood mononuclear cells (PBMCs) were isolated from ITP patients by gradient density centrifugation and cultured in complete RPMI‐1640 medium (Gibco, Grand Island, NY, USA) and 100 ng mL⁻^1^ of recombinant human macrophage colony‐stimulating factor (MCSF, PeproTech, Rocky Hill, NJ, USA) for 7 days. The adherent cells were considered as M0 macrophages and the purity of CD68^+^ macrophages was detected in more than 90% by flow cytometry (Figure S1A, Supporting Information). The M1 macrophages were induced by lipopolysaccharide (LPS, 20 ng mL⁻^1^, Sigma Aldrich, St. Louis, MO, USA) and interferon γ (IFNγ, 30 ng mL⁻^1^, PeproTech, Rocky Hill, NJ, USA) for 8 h and M2 macrophages were induced using IL4 (20 ng mL⁻^1^, PeproTech, Rocky Hill, NJ, USA) and IL13 (20 ng mL⁻^1^, PeproTech, Rocky Hill, NJ, USA) for 48 h. YC‐4‐3 or selective EIF2AK3/PERK activator CCT020312 (5 µm, Abmole, Houston, TX, USA) was added at the same time according to experimental grouping. Mouse bone marrow‐derived macrophages (BMDM) were cultured from a single cell suspension of the bone marrow of ITP murine models in the same manner. The mycoplasma testing (Yeason, Shanghai, China) was negative for each culture. The flow chart of cell culture was shown in Figure S1B (Supporting Information).

### 2.5 T Cell Co‐Culture

Macrophages from PBMCs of ITP patients were first cultured and treated as described above. Then media supernatant was replaced as complete RPMI‐1640 medium (Gibco, Grand Island, NY, USA) supplemented with anti‐human CD3 antibody (1 µg mL⁻^1^, Thermo Fisher Scientific, Waltham, MA, USA), anti‐human CD28 antibody (1 µg mL⁻^1^, Thermo Fisher Scientific, Waltham, MA, USA) and recombinant human IL‐2 (5 ng mL⁻^1^, R&D Systems, Minneapolis, MN, USA). CD4^+^ T cells isolated by magnetic activated cell sorting (Miltenyi Biotec GmbH, Bergisch Gladbach, Germany) from PBMCs of ITP patients were added concurrently at 10^5^ cells/mL. All cells were cultured for 3 days and then CD4^+^ T cells were prepared for flow cytometry.

### 2.6 Quantitative Real‐Time Polymerase Chain Reaction (q‐RTPCR) Analysis

RNA was extracted from macrophages using TRIZOL reagent (Takara, Tokyo, Japan) and reverse‐transcripted into cDNA using the cDNA synthesis kit (Yeason, Shanghai, China) according to the manufacturer's instructions. The mRNA expression of target genes was quantified by q‐RTPCR using the SYBR Premix Ex Taq (Yeason, Shanghai, China). Each sample was conducted as an independent experiment and assayed in triplicate, and the mean value was calculated as final results for each sample. Actin was set as internal inference. The primer sequences were seen in Table 2 (Supporting Information).

### 2.7 Flow Cytometry

PBMCs from healthy controls and ITP patients, as well as cultured macrophages and CD4^+^ T cells were stained with the corresponding antibodies (BioLegend, San Diego, CA, USA) according to the manufacturer's manual; detailed information of fluorescent antibodies is shown in Table 3 (Supporting Information). The acquisition was performed on a FACS AriIII flow cytometer (BD Biosciences, San Jose, CA, USA) and then analyzed using Flowjo software version 10.0.1. Each sample was conducted as an independent experiment and assayed in triplicate, and the mean value was calculated as final results for each sample.

### 2.8 Phagocytosis Assay

Phagocytosis of platelets was performed according to previously reported methods with mild modifications.^[^ ^36^, ^37^, ^38^ ^]^ Briefly, fresh platelets from ITP patients were labeled with CMFDA (Yeason, Shanghai, China) for 2 h at 37 °C in the presence of prostaglandin E1 (Abmole, Houston, TX, USA) and opsonized by anti‐CD41 monoclonal antibody (Thermo Fisher Scientific, Waltham, MA, USA) for 30 min at room temperature. Opsonized platelets were then incubated with macrophages for 1 h at 37 °C or 4 °C as control (platelets: macrophages = 50:1) and followed by quenching of extracellular fluorescence to 0.1% trypan blue (Solarbio, Beijing, China). The adherent macrophages were washed and digested, then stained with an anti CD61 APC antibody (BioLegend, San Diego, CA, USA) for flow cytometry. The mean fluorescence intensity (MFI) of CMFDA in CD61‐APC negative cells represented the engulfment of platelets by macrophages. The phagocytic index was calculated as the MFI obtained at 37 °C divided by the MFI at 4 °C. Phagocytosis of FITC‐IgG‐coated beads (Cayman Chemical, Ann Arbor, MI, USA) was performed according to the manufacturer's instructions. Briefly, macrophages were cocultured with beads at 1: 100 for 2 h at 37 °C, and then quenched for extracellular fluorescence with 0.1% trypan blue.

### 2.9 Co‐Immunoprecipitation (CO‐IP) and Liquid Chromatography Tandem Mass Spectrometry (LC‐MS/MS)

M0 Macrophages from ITP patients were lysed and the supernatant was incubated with anti GSK‐3β antibody (Abmart, Shanghai, China), and protein A/G magnetic beads (Sigma‐Aldrich, St. Louis, MO, USA) at 4 °C overnight according to the manufacturer's instruction. Then the beads were washed and collected for LC‐MS/MS and western blot. For LC‐MS/MS, the precipitate obtained in the CO‐IP experiment was desaturated, alkylated, digested to peptides, and desalted. The enzymatic hydrolysis products from two independent experiments were separated by capillary high performance liquid chromatography (HPLC) (Nano‐HPLC UltiMate 3000 RSLCnano, Thermo Fisher Scientific, Waltham, MA, USA) and subjected to mass spectrometry using a Q‐Exactive plus mass spectrometer (Thermo Fisher Scientific, Waltham, MA, USA). The acquired mass spectrometry data were searched by ProteomeDiscover 2.5 based on the human reviewed uniprot protein database. Overlapped proteins from the two samples were used for KEGG and GO enrichment analysis. The protein‐protein interaction (PPI) and Density of Maximum Neighborhood Component (DMNC) analysis were performed by Cytoscape (Version: 3.9.0).

### 2.10 Western Blot

Proteins extracted from macrophages and COS‐IP were collected, quantified, and denatured in sample buffer (Thermo Fisher Scientific, Waltham, MA, USA), subjected to SDS‐polyacrylamide gel electrophoresis (EpiZyme, Shanghai, China) and transferred to polyvinylidene fluoride membranes (Merck Millipore, Billerica, MA, USA). The specific antibodies (Table 4, Supporting Information) were used to specifically bind to proteins and for semi‐quantitatively analysis. β‐tubulin was used as the internal reference. Each sample was conducted as an independent experiment. The membranes were detected using ChemiScope Imaging System by auto‐exposure (Clinx, Shanghai, China).

### 2.11 Public Dataset Analysis

Six samples of murine macrophages in a dataset of GSE162464 from the Gene Expression Omnibus (GEO) database were selected to analyze the differentially expressed genes (DEGs),^[^ ^39^ ^]^ including three samples of control (non‐targeted) macrophages and three samples of sgGSK3b knockout macrophages generated using CRISPR in the absence of IFN‐γ stimulation. Differential genes were explored via the R package “limma”. The GO and KEGG enrichment analysis and gene list enrichment based on transcription factor targets were performed by Metascape (http://metascape.org/gp/index.html#/main/step1). ER stress‐related genes were considered as genes with a relevance score ≥ 7 extracted from GeneCards (https://www.genecards.org/).^[^ ^40^ ^]^ Venn plot and GSEA analysis were performed via the R packages “VennDiagram” and “clusterprofile”. The advanced volcano plot was performed using the OmicStudio tool at https://www.omicstudio.cn/tool.

### 2.12 Single‐Cell RNA Sequencing

PBMCs from three newly diagnosed ITP patients^[^ ^1^ ^]^ and three essential thrombocythemia (ET) patients^[^ ^41^ ^]^ according to the respective diagnostic criteria were collected for the single‐cell RNA sequencing by BD Rhapsody system. The single‐cell RNA Seq libraries were generated according to the manufacturer's protocol. Single‐cell RNA seq data of PBMCs from three samples of healthy controls were downloaded from GEO: GSE163668.^[^ ^42^ ^]^ The quality control, processing, correction of batch effects, and analysis for the gene matrices were performed by Seurat package (version 4.3.0.1),^[^ ^43^ ^]^ Harmony (version 0.1.1),^[^ ^44^ ^]^ and ggplot2 package (version 3.4.0)^[^ ^45^ ^]^ in R (version 4.3.1). Dimensionality reduction for the integrated dataset was performed using PCA, and Uniform Manifold Approximation and Projection (UMAP) plots were then generated. The signature scores for immune cell clusters were calculated using Ucell (2.4.0) in R.^[^ ^46^ ^]^ The cluster of monocytes is based on canonical marker genes according to previous reports.^[^ ^47^, ^48^ ^]^ Significant KEGG terms were enriched for each cluster using clusterProfiler package (version 4.8.3)^[^ ^49^ ^]^ in R with filtered DEGs.

### 2.13 Protein Docking

The 3D structures of human proteins HSPA5 (ID: AF‐P11021) and GSK‐3β (ID: AF‐P49841) were downloaded by AlphaFold 2. Protein‐protein docking between HSPA5 and GSK3B was performed using ZDOCK 3.0.2, followed by the selection of the optimal docking result. The PyMol V2.4.0 software was employed for plotting.

### 2.14 Statistical Analysis

All analyses were performed using GraphPad Prism 10.0. Correlation analysis was performed by Pearson correlation analysis. Data are expressed as the mean ± SD. Sample size for each statistical analysis was shown in corresponding figure legends. Normality was assessed by the Shapiro‐Wilk test. Pairwise comparison was analyzed by Student *t*‐test or Mann‐Whitney test based on normality. Multi‐group comparison was determined by one‐way ANOVA or Welch ANOVA as appropriate. Simple linear regression for linear correlation analysis. *P* values < 0.05 (two sides) were considered statistically significant.

### 2.15 Ethics Approval and Consent to Participate

The study was in accordance with the ethical standards formulated in the Helsinki Declaration and was approved by the Institutional Review Board of Zhongshan Hospital, Fudan University (approval No. B2020‐279R). Written informed consent was obtained from each participant included in the study.

## 3 Results

### 3.1 The Expression of GSK‐3β Was Elevated in ITP Patients and Decreased in Response Patients after Treatment

**Figure 1:**
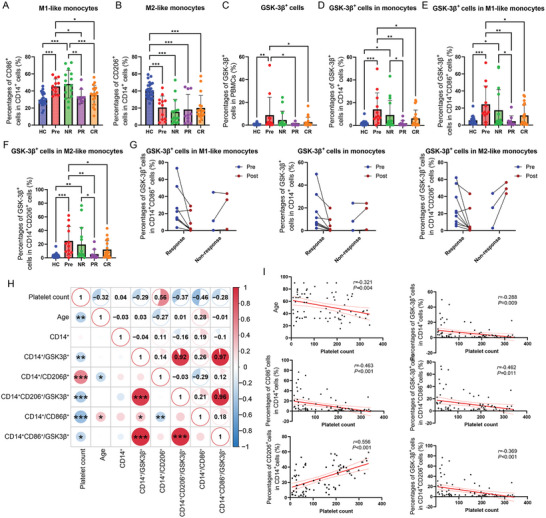
The expression of GSK‐3β in ITP patients and healthy controls. Peripheral blood mononuclear cells (PBMCs) from healthy controls and ITP patients were estimated by flow cytometry. A,B) The percentages of CD86^+^ M1‐like monocytes (A) and CD206^+^ M2‐like monocytes (B) CD14^+^ monocytes in healthy controls and ITP patients. C–F) The percentages of GSK‐3β^+^ cells in PBMCs (C), CD14^+^ monocytes (D), CD14^+^CD86^+^ M1‐like monocytes (E), and CD14^+^CD206^+^ M2‐like monocytes (F) in healthy controls and ITP patients. G) The percentages of GSK‐3β^+^ cells in newly diagnosed ITP patients before and after treatment. H) Correlation heat map. The upper part represented the correlation coefficients, and the lower half represented the *P* values of the correlation analysis. I) The linear regression analysis showed 95% confidence intervals. N = 26 for HC, N = 12 for Pre, N = 15 for NR, N = 10 for PR, N = 18 for CR. HC: healthy controls, Pre: Newly diagnosed ITP patients, NR: ITP patients with non‐response, PR: ITP patients achieving CR, CR: ITP patients achieving CR, CD14+/GSK3β^+^: the percentages of GSK‐3β^+^ cells in CD14^+^ cells, Error bars indicate mean ± SD. The statistical test is one‐way ANOVA with Tukey's multiple testing correction for A‐F, Pearson correlation analysis for H, simple linear regression for I.* *P* < 0.05, **P < 0.01, *** *P* < 0.001.

The percentages of CD14^+^CD86^+^ M1‐like monocytes and CD14^+^CD206^+^ M2‐like monocytes were analyzed in HCs and ITP patients (Figure S2, Supporting Information). The percentages of M1‐like monocytes were significantly increased in newly diagnosed ITP patients and non‐response ITP patients and decreased in ITP patients achieving PR and CR (**Figures** **1**A, S3A, Supporting Information). The percentages of M2‐like monocytes were significantly decreased in all ITP patients, even in patients achieving CR (Figure 1B; Figure S3B, Supporting Information). The percentages of GSK‐3β^+^ cells in PBMCs were determined to be expanded in newly diagnosed ITP patients and descended after effective treatment (Figure 1C; Figure S3C, Supporting Information). Then the expression of GSK‐3β was further analyzed in monocytes, newly diagnosed ITP patients and non‐response patients obtained higher GSK‐3β^+^ cells in CD14^+^ monocytes, while ITP patients achieving PR and CR have reduced GSK‐3β^+^ monocytes (Figure 1D; Figure S3D, Supporting Information). The similar changes were observed in M1‐like and M2‐like monocytes, the percentages of GSK‐3β^+^ cells were significantly proliferated in newly diagnosed and treatment non‐response patients compared with healthy controls and patients achieving PR and CR (Figure 1E,F; Figure S3E,F, Supporting Information). Among the 12 newly diagnosed ITP patients, nine patients were treatment‐responsive (PR+CR), and three patients showed non‐response to treatment. Most of the treatment‐response ITP patients showed significantly diminished GSK‐3β^+^ cells in all monocytes, M1‐like monocytes and M2 monocytes, while treatment non‐response patients presented increased or slightly changed GSK‐3β^+^ cells (Figure 1G). The correlation analysis and linear regression analysis showed that the platelet count was negatively correlated with age, GSK‐3β^+^ cells in CD14^+^ monocytes, GSK‐3β^+^ cells in M1‐like and M2‐like monocytes and CD86^+^ M1‐like cells, and was positively correlated with M2‐like monocytes (Figure 1H,I).

### 3.2 YC‐4‐3 Alleviated the CD41 Antibody‐Induced Thrombocytopenia

**Figure 2:**
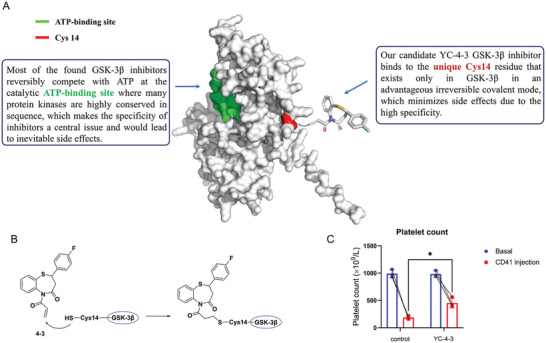
The binding sites of GSK‐3β inhibitors and effects on thrombocytopenia. A) The structure of the GSK‐3β and binding sites of GSK‐3βi. B) The structural formula showed how the novel GSK‐3βi acted on the effective structural domain of GSK‐3β. C) Platelet count of mice. The WT C57BL/6 mice were treated with the novel GSK‐3βi at 5 mg kg⁻^1^ d⁻^1^ or equiv. DMSO for 3 days, and received anti‐mouse CD41 monoclonal antibody injection intraperitoneally at dose of 68 µg kg⁻^1^. Platelet count was measured on the second day after anti‐mouse CD41 monoclonal antibody injection, error bars indicate mean ± SD, N = 3 for each group, Mann‐Whitney test, * *P* < 0.05.

The novel GSK‐3βi binds to the unique Cys14 residue that exists only in GSK‐3β in an advantageous irreversible covalent mode, while most of the found GSK‐3βi reversibly compete with ATP at the catalytic ATP‐binding site (**Figure** **2**A,B). After being treated with the novel GSK‐3βi at 5 mg/ kg/d for 3 days, the WT C57BL/6 mice received anti‐mouse CD41 monoclonal antibody injection intraperitoneally at a dose of 68 µg kg⁻^1^. The platelet counts were examined on the second day and the results showed that mice receiving YC‐4‐3 treatment had a milder reduction in platelet counts than control mice (Figure 2C), suggesting the potential therapeutic value of YC‐4‐3 in anti‐platelet antibody‐mediated thrombocytopenic diseases.

### 3.3 YC‐4‐3 Affected the Polarization and Function of Macrophages In Vitro

**Figure 3:**
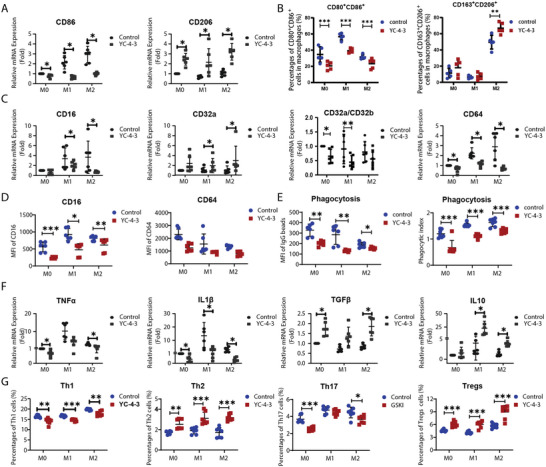
The effects of GSK‐3βi on macrophages in vitro. Macrophages derived from PBMCs (peripheral blood mononuclear cells) of immune thrombocytopenia (ITP) patients were treated with 10 µm GSK‐3βi. A) Relative mRNA expression of CD86 and CD206 in macrophage (N = 6). B) Percentages of CD80^+^CD86^+^ M1 macrophages and CD163^+^CD206^+^ M2 macrophages in CD68^+^ macrophages (N = 6). C) Relative mRNA expression of CD16, CD32a, CD32a/CD32b, and CD64 in macrophages (N = 6). D) MFI of CD16 and CD64 in CD68^+^ macrophages (N = 6). E) MFI of FITC‐IgG‐coated beads in macrophages (Left) (N = 6). Phagocytic index of platelets in macrophages (Right) (N = 6). F) Relative mRNA expression of IL1β, TNFα, IL10, and TGFβ in macrophages (N = 6). G) Percentages of Th1, h2, Th17, and Tregs in CD4^+^ T cells (N = 7). Error bars indicate mean ± SD, Mann‐Whitney test, * *P* < 0.05, **P < 0.01, *** *P* < 0.001.

The viability of M0 macrophages induced from PBMCs of ITP patients was assessed in the presence of different concentrations of GSK‐3βi cells that had comparable high viability at the concentration of no more than 25 µm (Figures S4 and S5A, Supporting Information). For polarization of macrophages, YC‐4‐3 significantly decreased the expression of CD80 and CD86 and enhanced the expression of CD163 and CD206 at mRNA (**Figure** **3**A; Figure S5B, Supporting Information) levels. YC‐4‐3 decreased the percentages of CD80^+^CD86^+^ M1 macrophages and increased the percentages of CD163^+^CD206^+^ M2 macrophages (Figure 3B; Figure S5C,D, Supporting Information). For Fcγ receptors mediated phagocytosis, YC‐4‐3 reduced the expression of CD16 and CD64, and decreased the ratio of CD32a/CD32b (Figure 3C,D; Figure S6A,B, Supporting Information). YC‐4‐3 inhibited the phagocytic capacity of platelets and IgG‐coated beads in M0, M1, and M2 macrophages (Figure 3E; Figure S6C, Supporting Information). For the regulation of the immune response, YC‐4‐3 weakened the generation of inflammatory cytokines IL1β and TNFα, and amplified the anti‐inflammatory cytokines IL10 and TGFβ (Figure 3F). Since YC‐4‐3 showed a strong immunomodulatory effect at 10 µm with minimal influence on cell survival in vitro, 10 µm was chosen as the optimal concertation for experiments in vitro. For the modulation of T cells, YC‐4‐3 treated macrophages promoted the differentiation of Th cells to Th2 and Tregs and inhibited the differentiation of Th1 and Th17 cells (Figure 3G; Figure S6D–F, Supporting Information).

### 3.4 YC‐4‐3 Alleviated Thrombocytopenia and Modulated Macrophages in ITP Mice

**Figure 4:**
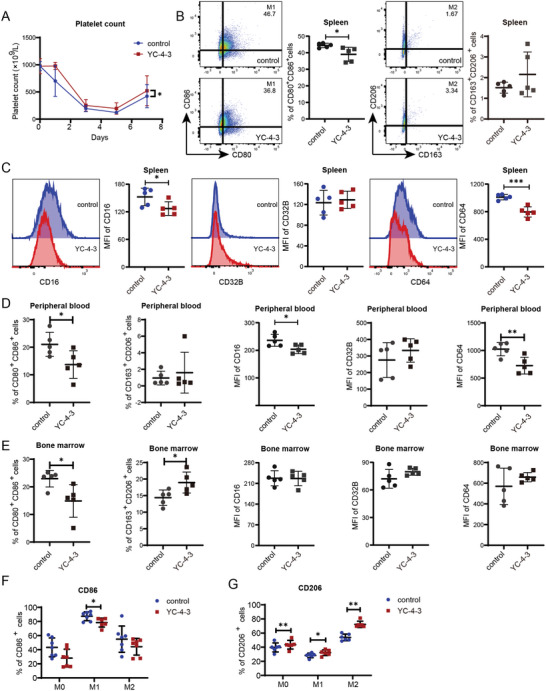
The effects of GSK‐3βi on macrophages in passive ITP mice models. Passive ITP murine models were established and treated with or without YC‐4‐3 (N = 5). A) Platelet count of mice in YC‐4‐3 group and control group. B) Percentages of CD80^+^CD86^+^ M1 macrophages and CD163^+^CD206^+^ M2 macrophages in CD11b^+^F4/80^+^ macrophages in the spleen. Flow cytometry dot plots showed the gate for M1 and M2 macrophages. C) MFI of CD16, CD32B, and CD64 in CD11b^+^F4/80^+^ macrophages in the spleen. Histogram plots showed the fluorescence intensity in flow cytometry. D,E) Percentages of CD80^+^CD86^+^ and CD163^+^CD206^+^ macrophages and MFI of CD16, CD32b, and CD64 in CD11b^+^F4/80^+^ macrophages in D) peripheral blood and E) bone marrow. F,G) Bone marrow‐derived macrophages from passive ITP mice were treated with 10 µm GSK‐3βi in vitro. Percentages of F) CD86^+^ M1 macrophages and G) CD206^+^ M2 macrophages in CD11b^+^F4/80^+^ BMDM. Error bars indicate mean ± SD, t test, * *P* < 0.05, **P < 0.01, *** *P* < 0.001.

In the passive murine ITP model, the platelet counts of YC‐4‐3 group recovered more rapidly than the control group during the process of model induction (**Figure** **4**A). YC‐4‐3 significantly decreased the percentage of CD80^+^CD86^+^ M1 macrophages and the expression of CD16 and CD64 on macrophages in the spleen (Figure 4B,C) and peripheral blood (Figure 4D). In bone marrow, YC‐4‐3 significantly decreased the percentages of CD80^+^CD86^+^ M1 macrophages and increased CD163^+^CD206^+^ M2 macrophages (Figure 4E). YC‐4‐3 also reduced the percentages of CD86^+^ macrophages and increased CD206^+^ macrophages in BMDM from ITP mice (Figure 4F,G).

**Figure 5:**
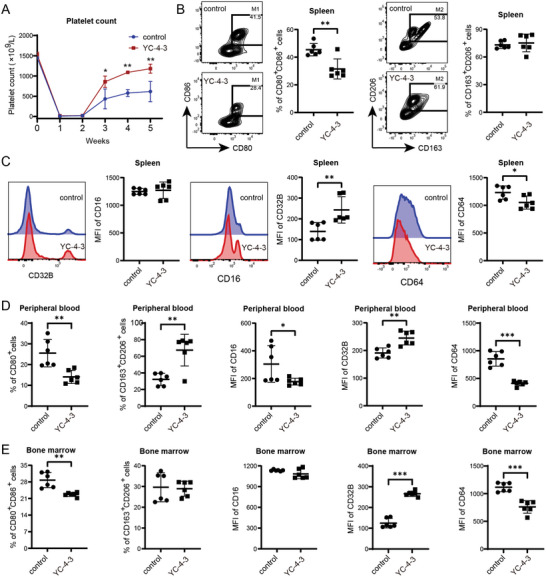
The effects of GSK‐3βi on macrophages in active ITP mice models. Active ITP murine models were established and treated with or without YC‐4‐3 (N = 6). A) Platelet count of mice in YC‐4‐3 group and control group. B) Percentages of CD80^+^CD86^+^ M1 macrophages and CD163^+^CD206^+^ M2 macrophages in CD11b^+^F4/80^+^ macrophages in the spleen. Flow cytometry density plots showed the gate for M1 and M2 macrophages. C) MFI of CD16, CD32B, and CD64 in CD11b^+^F4/80^+^ macrophages in the spleen. Histogram plots showed the fluorescence intensity in flow cytometry. D,E) Percentages of CD80^+^CD86^+^ and CD163^+^CD206^+^ macrophages and MFI of CD16, CD32b, and CD64 in CD11b^+^F4/80^+^ macrophages in D) peripheral blood and E) bone marrow. Error bars indicate mean ± SD, t test, * *P* < 0.05, **P < 0.01, *** *P* < 0.001.

In the active ITP model, YC‐4‐3 began to significantly increase the platelet count in the third week, and this effect sustained until the fifth week (**Figure** **5**A). Similar to the passive ITP mice, YC‐4‐3 significantly decreased the percentage of CD80^+^CD86^+^ M1 macrophages (Figure 5B), repressed the expression of CD64, and increased CD32B on macrophages in the spleen (Figure 5C) and bone marrow (Figure 5E). In peripheral blood, YC‐4‐3 significantly decreased the percentage of CD80^+^CD86^+^ M1 macrophages and the expression of CD16 and CD64 on macrophages, and decreased the CD163^+^CD206^+^ M2 macrophages and CD32B expression (Figure 5D).

### 3.5 YC‐4‐3 Impacted PI3K/mTOR/Akt, MAPK, and NFκB/IκBα Pathways in Macrophages

**Figure 6:**
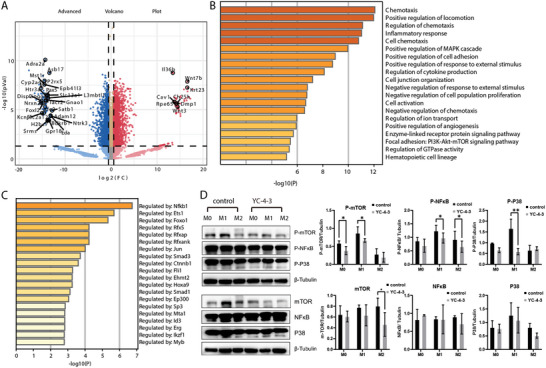
Gene functional enrichment analysis and validation of dataset. Differential gene analysis and gene functional enrichment analysis of GSE162464 dataset. A) Volcano plots for differentially expressed genes (DEGs) in GSE162464 (*P* < 0.05, fold change > 1.5). B) GO‐KEGG Enrichment analysis, colored by *p*‐values using Metascape. C) Summary of enrichment analysis in Transcription Factor Targets based on Metascape. D) Western blot of the main proteins involved in PI3K/mTOR/Akt, MAPK, and NFκB/IκBα pathways. The bar plots showed the relative gray values, results from four independent experiments. * *P* < 0.05, ** *P* < 0.01.

To elucidate the potential mechanisms of GSK‐3β in macrophages, a public database consisting of GSK‐3β knockout and control macrophages without stimulation was used to map the transcriptome. A total of 3 468 genes showed significant differences (adj.*P* < 0.05, fold change > 1.5), of which 1 798 genes were up‐regulated and 1 670 genes were down‐regulated in the GSK‐3β knockout group compared to the control group (**Figure** **6**A). KEGG‐GO pathway enrichment analysis showed the participation of the MAPK cascade, the PI3K‐Akt‐mTOR signaling pathway, and several pathways related to inflammatory responses (Figure 6B). The transcription factor enrichment analysis showed the regulation of Nfkb1, which is a transcription factor in the NFκB pathway (Figure 6C). The signaling pathways enriched from the database were verified by western blot in macrophages derived from ITP patients, the results showed that YC‐4‐3 inhibited the phosphorylation of proteins in PI3K/mTOR/Akt, MAPK and NFκB/IκBα pathways (Figure 6D; Figure S7A–E, Supporting Information).

### 3.6 YC‐4‐3 Restrained ER Stress in Macrophages of ITP

**Figure 7:**
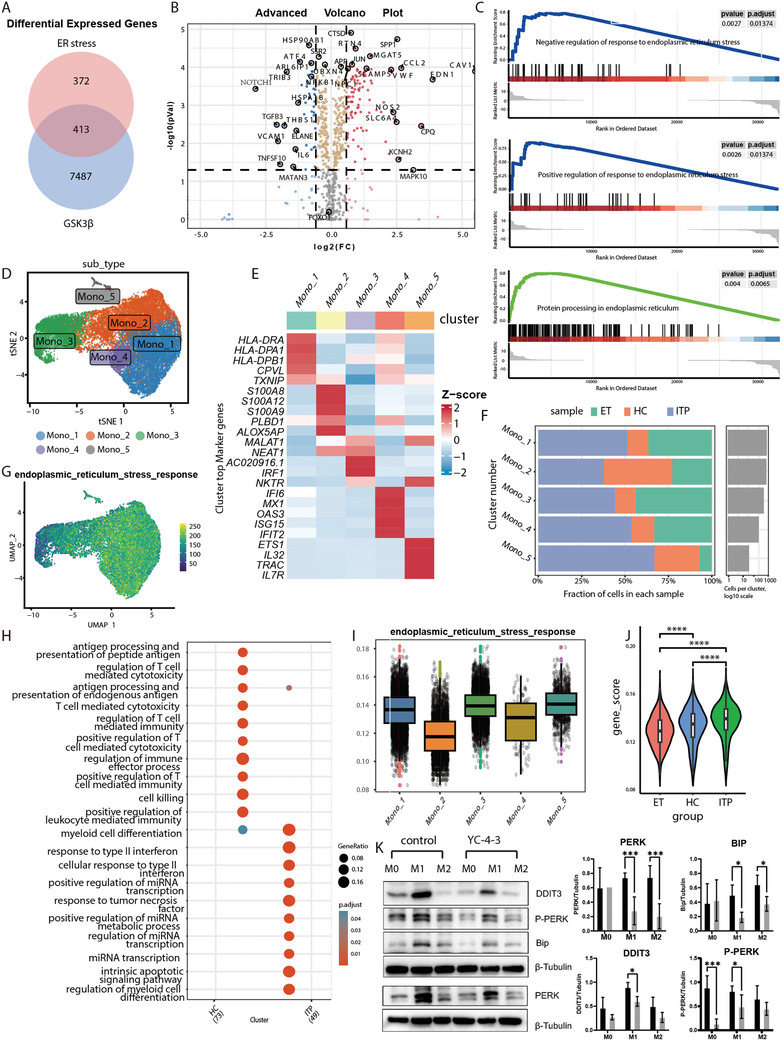
Gene functional enrichment analysis and validation of the ER stress pathway. Functional enrichment analysis of genes in GSE162464 dataset and genes related with ER stress and single cell RNA‐Seq analysis of monocytes in healthy controls (HC), immune thrombocytopenia (ITP), and essential thrombocythemia (ET) patients. A) Venn plot showed the intersection genes between DEGs (*P* < 0.05) in GSE162464 dataset and ER stress related genes. B) Volcano plots for ER stress related genes (P < 0.05, fold change > 1.5). C) GSEA plots based on GO (the top two plots) and KEGG (the bottom plot) enrichment analysis. D) UMAP plot for single cell RNA‐Seq of total monocytes from HC, ITP, and ET patients. E) Heap map plot for the expression of key genes used for cluster identification, grouped by monocyte subset cluster. F) Fraction of monocyte subsets in HC, ITP, and ET patients. G) UMAP visualization of gene expression of ER stress pathway in monocytes. H) KEGG enrichment of monocytes in ITP patients and HC. I) Relative gene expression of ER stress in different subsets of all the monocytes from HC, ITP, and ET patients. J) Relative gene expression of ER stress in all the monocytes from HC, ITP, and ET patients. K) Western blot plot of the main proteins involved in ER stress pathways. The bar plots showed the relative gray values, results from four independent experiments. * *P* < 0.05, *** *P* < 0.001, **** *P* < 0.001.

There were 413 overlapped genes between 7 900 DEGs (adj.*P* < 0.05) in the GSK‐3β knockout dataset and 785 ER stress‐related genes detected by Venn analysis (**Figure** **7**A). Among all 754 ER stress‐related genes, there were a total of 136 genes showing significant differences (adj.*P* < 0.05, fold change > 1.5), of which 94 genes were up‐regulated and 42 genes were down‐regulated in the GSK‐3β knockout group compared to the control group (Figure 7B). Based on GSEA, GO analysis was significantly enriched in negative regulation of response to ER stress and positive regulation of response to ER stress, and KEGG analysis was significantly enriched in protein processing in the ER (Figure 7C). Sigle‐cell RNA sequencing of PBMCs from HC, ITP patients and ET patients presented five subsets of monocytes by UMAP clustering (Figure 7D). These clusters could be categorized into the three major monocyte subtypes by distinct gene expression patterns based on previous reports^[^ ^47^, ^48^ ^]^: Mono_1, Mono_3, and Mono_5 as intermediate monocytes, Mono_2 as classical monocytes and Mono_4 as non‐classical monocytes (Figure 7E). ITP patients showed higher fractions of Mono_1, 3, 4, and 5, and lower fraction of Mono_2 compared with HC and ET patients (Figure 7F). The KEGG enrichment analysis of monocytes from HC and ITP patients was performed (Figure 7H), and the expression of ER stress response pathway was visualized on UMAP clustering of monocytes (Figure 7G). The Mono_1, Mono_3, and Mono_5 displayed the higher expression of ER stress response pathway and Mono_2 presented the lowest expression (Figure 7I). Monocytes from ITP patients exhibited higher expression of ER stress response pathway than HC and ET patients (Figure 7J). To verify the influence of YC‐4‐3 on ER stress, proteins of macrophages derived from ITP patients were assessed by western blot. Results showed that the phosphorylation and synthesis of PERK, DDIT3, and Bip were inhibited by YC‐4‐3 (Figure 7K; Figure S6F–J, Supporting Information).

### 3.7 The Intervention of ER Stress Affected the Modulation of YC‐4‐3 in Macrophages

**Figure 8:**
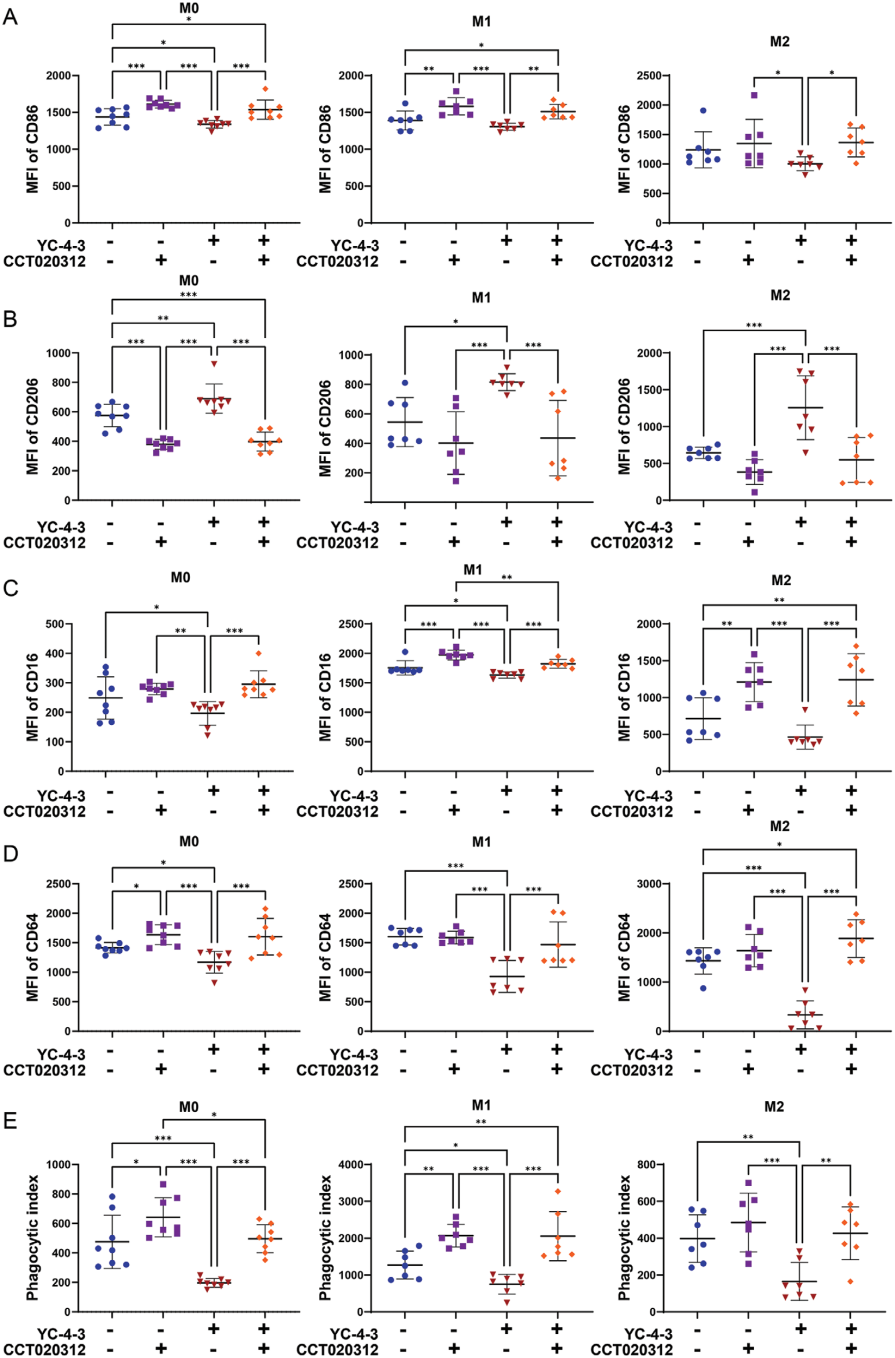
The effects of intervening ER stress and GSK‐3β on macrophages in vitro. Macrophages derived from PBMCs of ITP patients were treated with or without the YC‐4‐3 (10 µm), selective EIF2AK3/PERK activator CCT020312 (5 µm). A–D) MFI of CD86 (A), CD206 (B), CD16 (C), and CD64 (D) in M0, M1, and M2 macrophages under different treatments. E) The phagocytic index in M0, M1, and M2 macrophages under different treatments. N = 8 for M0 macrophages, N = 7 for M1 and M2 macrophages. Error bars indicate mean ± SD, t test, * *P* < 0.05, **P < 0.01, *** *P* < 0.001.

As a selective EIF2AK3/PERK activator, CCT020312 significantly increased the expression of CD86, the Fcγ receptor, CD16 and CD64, and the phagocytosis of platelets in macrophages, and decreased the expression of CD206 when compared with controls (**Figure** **8**A–E). Compared with YC‐4‐3 treated cells, the additional supplement of CCT020312 in YC‐4‐3 treated macrophages significantly increased the expression of CD86, CD16, CD64, and the phagocytosis of platelets, and decreased the expression of CD206 (Figure 8A–E).

### 3.8 GSK‐3β Combines with Bip in Macrophages

**Figure 9:**
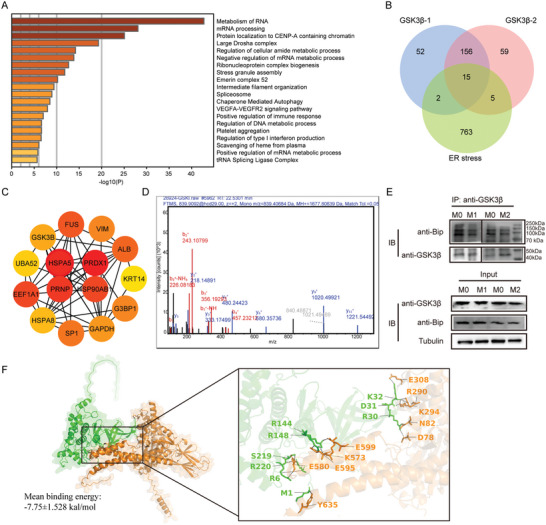
Co‐immunoprecipitation (CO‐IP) and liquid chromatography‐tandem mass spectrometry (LC‐MS/MS) for GSK‐3β. Proteins combined with GSK‐3β were precipitated and analyzed by LC‐MS/MS. A) GO‐KEGG Enrichment analysis of coding genes for CO‐IP obtained proteins, colored by p‐values using Metascape. B) Venn plot for overlapping genes among two samples of CO‐IP protein coding genes and ER stress related genes. C) Protein‐protein interaction (PPI) network of 15 overlapping proteins. The color intensity of each node was proportional to the Maximum Neighborhood Component (DMNC) degree. D) LC‐MS/MS base peak chromatogram of Bip. E) Western blot plot for CO‐IP of GSK‐3β. The lanes of M0/M1 and M0/M2 were from separative membranes. IB: immunoblotting, IP: Immunoprecipitation. F) Protein docking of GSK3β and Bip. The right plot showed the binding sites of GSK3β with Bip. Bip protein is depicted in orange, GSK‐3β protein is shown in green. Dashed lines represent hydrogen bonds indicating amino acid residue interactions. The capital letters represent the abbreviations of the corresponding amino acids, and the numbers indicate the positional information of these amino acids within the protein sequence.

A total of 171 interacting proteins from two samples were identified from CO‐IP experiments by LC‐MS/MS analysis. KEGG and GO enrichment analysis classified the detected proteins by their biological function, and the results revealed that these proteins were closely related to the metabolism and processing of mRNA and protein (**Figure** **9**A). There were 15 overlapped protein coding genes among CO‐IP protein‐coding genes and ER stress‐related genes (Figure 9B). The interactions of these proteins were revealed by PPI network, among which HSPA5 (also known as GRP78 and BiP, Organism: Homo sapiens, NCBI Reference Sequence: NP_0 05338.1) had the highest DMNC score and was tightly related to ER stress (Figure 9C). LC‐MS/MS base peak chromatograms showed the detection of Bip (Figure 9D). The interactions of GSK‐3β and Bip in macrophages were confirmed by CO‐IP (Figure 9E). Molecular docking was performed to investigate the interactions between GSK‐3β and Bip, which revealed a strong binding affinity with a mean binding energy of ‐7.75 ± 1.528 kcal mol⁻^1^ for the Bip‐GSK‐3β complex (Figure 9F). Further analysis of the binding site indicated that the binding of Bip to GSK‐3β was stabilized by hydrogen bond interactions with the amino acid residues *Glu* 308, *Arg* 290, *Lys* 294, *Asn* 82, *Asp* 78, *Glu* 599, *Lys* 573, *Glu* 580, *Glu* 595 and *Tyr* 635 in Bip.

## 4 Discussion

GSK‐3β has impact on macrophage polarization, migration, and proliferation; However, the role of GSK‐3β in ITP patients has been seldom studied. In this study, the anomalous increased GSK‐3β^+^ cells in newly diagnosed ITP patients were measured, especially in M1‐like and M2‐like monocytes of patients, which can be rectified by effective HD‐DXM treatment, suggesting that GSK‐3β plays an important role in the progression of disease. And our data showed that there is negative correlation between GSK‐3β expression and platelet count, and the negative correlation of GSK‐3β expression and M2‐like monocytes, indicating the participation of GSK‐3β in regulating pro‐inflammatory response in mononuclear‐macrophage system, and the inhibition of GSK‐3β may alleviate the disease.

Given the central role of macrophages in ITP, targeting macrophages has emerged as an important way for disease treatment,^[^ ^38^ ^]^ including traditional treatment such as glucocorticoid,^[^ ^18^, ^19^, ^37^ ^]^ intravenous immunoglobulin (IVIG)^[^ ^50^ ^]^ and thrombopoietin receptor agonists (TPO‐RA),^[^ ^51^ ^]^ and novel pharmaceuticals such as Syk inhibitors and CD38+ antibody.^[^ ^15^, ^52^, ^53^ ^]^ Consistent with previous researches, our data showed that the increased fractions of intermedial monocytes and non‐classical monocytes in newly diagnosed ITP patients, accompanied by decrease in classical monocytes by single‐cell RNA seq.^[^ ^54^, ^55^ ^]^ Based on the results of the correlations between GSK‐3β and monocytes in ITP patients, inhibiting GSK‐3β could exert therapeutic effects by regulating macrophages in ITP. Most of the found GSK‐3βi reversibly compete with ATP at the catalytic ATP‐binding site where many protein kinases are highly conserved in sequence, which makes the specificity of the inhibitors a central issue and would lead to inevitable side effects. Our patented GSK‐3βi (YC‐4‐3) binds to the unique Cys14 residue that exists only in GSK‐3β in an advantageous irreversible covalent mode, which minimizes side effects due to the high specificity. The YC‐4‐3 could regulate the differentiation, phagocytosis, and cytokine generation of macrophages in ITP, and exhibit a satisfactory therapeutic effect on thrombocytopenia in either passive or active ITP murine models. Pharmacological Inhibition of GSK‐3β could attenuate the PI3K/mTOR/Akt, NFκB/IκBα, and MAPK pathways,^[^ ^56^ ^]^ which are related to the inflammatory responses in macrophages. These results provide evidence for the application of GSK‐3β as a therapeutic agent in ITP.

The transcriptional and protein levels were analyzed to provide the mechanisms of GSK‐3β in regulating macrophages. ER stress is caused by the disturbed synthesis and secretion of the protein, links inflammation, metabolic signals, and other cellular processes via the unfolding protein response (UPR).^[^ ^24^ ^]^ Increased gene levels of ER stress pathways in monocytes from ITP patients than that from healthy individuals were found. Previous studies reported that the activity of GSK‐3β is positively related with PERK, the ER stress response sensor;^[^ ^20^, ^23^, ^57^, ^58^ ^]^ However, there are few studies on the mechanism of GSK‐3 regulation of ER stress. The data of this study suggested that inhibition of GSK‐3β could alleviate ER stress. Pharmacological activation of PERK attenuates the phenotypic changes of macrophages induced by GSK‐3βi. ER stress might be the downstream signaling pathway linking GSK‐3β to the function of macrophages. In addition, GSK‐3β could directly interact with ER stress by binding to Bip. And the protein docking showed a strong binding ability between Bip and GSK‐3β. The protein chaperone, Bip, is one of the most abundant proteins in the ER. Bip is also thought to be a direct ER stress sensor that activates UPR.^[^ ^59^ ^]^ These results indicate that GSK‐3β could directly regulate the immune response of macrophages in combination with the ER stress response sensor.

**Figure 10:**
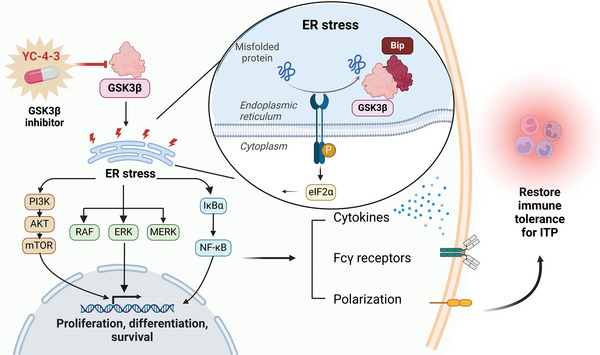
Graphic abstract GSK‐3βi regulates macrophages by alleviating ER stress in ITP. GSK‐3βi YC‐4‐3 could impede the ER stress signals by directly combining with the protein chaperone Bip, then suppressed the downstream PI3K/mTOR/Akt, NFκB/IκBα, and MAPK pathways and further restrained the pro‐inflammatory differentiation, phagocytosis, and cytokine generation of macrophages in ITP. Thus, GSK‐3βi could modulate the inflammatory status of macrophages and exert the therapeutic effects in ITP.

In conclusion, the present study revealed the aberrantly elevated expression of GSK‐3β^+^ monocytes in ITP patients, and the negative correlation between GSK‐3β and platelet counts. For the first time, our data showed that YC‐4‐3, a novel GSK‐3βi, could modulate the inflammatory status of macrophages in ITP in vitro and in vivo, and dampen the thrombocytopenia in both passive and active murine ITP models. Furthermore, it is found that the ER stress is the downstream signal of GSK‐3β. The study also identified a direct connection between GSK‐3β and ER stress that GSK‐3β could combine with an ER stress sensor Bip (**Figure** **10**). These findings provide a substantial body of evidence for a novel therapeutic strategy in ITP, that the inhibition of GSK‐3β could modulate the polarization and function of macrophages in ITP, and provide a novel irreversible covalent GSK‐3βi as a potential medicine for ITP.

## 5 Conclusions

Our data suggested that, for the first time, this new patented GSK‐3βi, new benzothiazepinone compounds (BTZs), YC‐4‐3, could modulate the inflammatory status of macrophages in ITP in vitro and in vivo, and improve the thrombocytopenia in both passive and active murine ITP models. The data also showed that ER stress is the downstream signal of GSK‐3β and could be the intersection of GSK‐3β and PI3K/mTOR/Akt, NFκB, and MAPK pathways. The connection between GSK‐3β and ER stress was identified that GSK‐3β could combine with an ER stress sensor Bip. Our findings provide a novel irreversible covalent GSK‐3βi, YC‐4‐3, as a potential medicine for ITP, which is substantial evidence for a novel therapeutic strategy in ITP.

## Conflict of Interest

The authors declare no conflict of interest.

## Author Contributions

Pengcheng Xu, Yunfeng Cheng, Yong Chu, Lili Ji, Yanxia Zhan, and Hao Chen performed the literature review, drafted and revised the manuscript. Yunfeng Cheng, Hao Chen, and Yong Chu contributed to the critical revision of the manuscript. Yanxia Zhan, Lili Ji, Hao Chen, Yang Ou, Xibing Zhuang, Xia Shao, Fanli Hua, Feng Li, and Yunfeng Cheng performed the experiments and analyzed the data. All authors read and approved the final manuscript.

## Supporting information



Supporting Information

## Data Availability

The data that support the findings of this study are available from the corresponding author upon reasonable request.
