# Supplementary material for: YC‐4‐3, a Novel Glycogen Synthase Kinase 3β Inhibitor, Alleviates the Endoplasmic Reticulum Stress of Macrophages in Primary Immune Thrombocytopenia
Source: Adv Sci (Weinh). 2025 Mar 7;12(17):2412515. doi: 10.1002/advs.202412515 (PMC12061250; doi:10.1002/advs.202412515)

**Supplemental Information**

A novel Glycogen synthase kinase 3β inhibitor regulates the polarization and function of macrophages by interrupting endoplasmic reticulum stress in immune thrombocytopenia

**This file includes:**

Supplementary table 1: Basic characteristics of enrolled individuals

Supplementary table 2: Primer sequences of target markers

Supplementary table 3: Fluorescence channels for flow cytometry

Supplementary figure 1: The purity of macrophages and procedure of cell culture.

Supplementary figure 2: The flow cytometry gates of peripheral blood mononuclear cells.

Supplementary figure 3: The GSK-3β^+^ cells and monocytes in ITP patients and healthy controls.

Supplementary figure 4: The effects of GSK-3β inhibitor on polarization of macrophages in vitro

Supplementary figure 5: The effects of GSK-3β inhibitor on the proinflammatory and phagocytosis functions of macrophages in vitro

Supplementary figure 6: The original figures of western blot

**Supplementary table 1.** **Basic characteristics of enrolled individuals**

|  | Healthy control (N=26) | ITP patients (N=43) | | |
| --- | --- | --- | --- | --- |
| Baseline statement | Healthy control | Newly diagnosed | Response | Non-response |
| Number | 26 | 12 | 19 | 12 |
| **Age (years),**  **mean (range)** | 53 (21-75) | 52 (20-80) | 56 (16-75) | 53 (15-80) |
| **Platelet count (×10^9^/L), mean (range)** | 250 (186-342) | 20 (8-27) | 40 (8-59) | 133 (56-331) |

Response: ITP patients achieving partial response and complete response.

## Supplementary table 2. Primer sequences of target markers

| **Gene** | **Accession number** | **Forward Primer** | **Reverse Primer** |
| --- | --- | --- | --- |
| **human** | | | |
| ACTB | NM_001101.5 | CATGTACGTTGCTATCCAGGC | CTCCTTAATGTCACGCACGAT |
| CD80 | NM_005191.4 | AAACTCGCATCTACTGGCAAA | GGTTCTTGTACTCGGGCCATA |
| CD86 | NM_001206925.2 | CTGCTCATCTATACACGGTTACC | GGAAACGTCGTACAGTTCTGTG |
| CD163 | XM_054373862.1 | GCGGGAGAGTGGAAGTGAAAG | GTTACAAATCACAGAGACCGCT |
| MRC1 | NM_002438.4 | GGGTTGCTATCACTCTCTATGC | TTTCTTGTCTGTTGCCGTAGTT |
| IL1B | XM_047444175.1 | TTCGACACATGGGATAACGAGG | TTTTTGCTGTGAGTCCCGGAG |
| IL10 | NM_001382624.1 | TCAAGGCGCATGTGAACTCC | GATGTCAAACTCACTCATGGCT |
| TNF | NM_000594.4 | ATGTTGTAGCAAACCCTCAAGC | GGACCTGGGAGTAGATGAGGT |
| TGFB1 | NM_000660.7 | CGTGGAGCTGTACCAGAAAT | TGAACCCGTTGATGTCCACTT |
| FCGR3A | XM_047449444.1 | AAGCCACACTCAAAGACAGC | ATGGTTGACACTGCCAAACC |
| FCGR2A | XM_054335138.1 | ATCATTGTGGCTGTGGTCATTG | TGTTTCATAGTCATTGTTGGTTTCTTC |
| FCGR2B | FCGR2B | ATTCCTGGCTCCTGTTGCTG | GAATGGAGTCGCTCTCAGGG |
| FCGR1A | NM_000566.4 | AGAAGAACAGCTGCAGGAAGG | TGGATGTTTGTACGCAGTGC |
| **Mouse** | | | |
| Actb | NM_007393.5 | CGTTGACATCCGTAAAGACC | TAGGAGCCAGAGCAGTAATC |
| Cd80 | XM_036159758.1 | ACCCCCAACATAACTGAGTCT | TTCCAACCAAGAGAAGCGAGG |
| Cd86 | XM_011245812.3 | TGTTTCCGTGGAGACGCAAG | TTGAGCCTTTGTAAATGGGCA |
| Cd163 | XM_006506800.5 | CTGGCGGGTGGTGAAAACA | CAGCCGTTACTGCACACTG |
| Mrc1 | NM_008625.2 | CTCTGTTCAGCTATTGGACGC | CGGAATTTCTGGGATTCAGCTTC |
| Il1b | XM_006498795.5 | CTGGTACATCAGCACCTCAC | AGAAACAGTCCAGCCCATAC |
| Il10 | XM_036162094.1 | CTTACTGACTGGCATGAGGATCA | GCAGCTCTAGGAGCATGTGG |
| Tnf | NM_001278601.1 | CAGGCGGTGCCTATGTCTC | CGATCACCCCGAAGTTCAGTAG |
| Tgfb1 | NM_011577.2 | ACAGGGCTTTCGATTCAGCG | GGAAGGGCCGGTTCATGTC |
| Fcgr3 | NM_001405018.1 | CAGAATGCACACTCTGGAAGC | GGGTCCCTTCGCACATCAG |
| Fcgr2b | NM_001419006.1 | AGGGCCTCCATCTGGACTG | GTGGTTCTGGTAATCATGCTCTG |
| Fcgr1 | NM_010186.5 | AGGTTCCTCAATGCCAAGTGA | GCGACCTCCGAATCTGAAGA |

## Supplementary table 3. Fluorescence channels for flow cytometry

| **Human** | | | | | |
| --- | --- | --- | --- | --- | --- |
| **Markers** | **Fluorescence channels** | **Lot number** | **Clone number** | **Catalogue number** | **Manufacturer** |
| CD14 | APC/CY7 | B396557 | M5E2 | 301820 | Biolegend |
| CD206 | Percp/cy5.5 | B385522 | 15-2 | 321122 | Biolegend |
| CD86 | APC | B396928 | BU63 | 374208 | Biolegend |
| GSK-3β | PE | 72115S | D5C5Z | 2 | CST |
| CD68 | APC | B407070 | Y1/82A | 333810 | Biolegend |
| CD86 | Percp/cy5.5 | B365534 | IT2.2 | 305420 | Biolegend |
| CD206 | PE | B375249 | 15-2 | 321106 | Biolegend |
| CD163 | PE/CY7 | B374568 | GHI/61 | 333614 | Biolegend |
| CD64 | BV421 | B431828 | 10.1 | 305020 | Biolegend |
| CD4 | FITC | B401363 | RPA-T4 | 300506 | Biolegend |
| IL4 | APC/CY7 | B409128 | MP4-25D2 | 500834 | Biolegend |
| IL17 | BV421 | B379495 | BL168 | 512322 | Biolegend |
| INFγ | Percp/cy5.5 | B373056 | 4S.B3 | 502526 | Biolegend |
| CD25 | APC | B384461 | BC96 | 302610 | Biolegend |
| FoxP3 | PE/CF594 | 2249441 | 259D/C7 | 562421 | BD Bioscience |
| Fiaxble viability stain | BV510 | B425259 |  | 423108 | Biolegend |
| **Mice** | | | | | |
| **Markers** | **Fluorescence channels** | **Lot number** | **Clone number** | **Catalogue number** | **Manufacturer** |
| F4/80 | PE | B423058 | BM8 | 123110 | Biolegend |
| CD11B | FITC | B369619 | M1/70 | 101205 | Biolegend |
| CD86 | PF/DAZZLE594 | B353566 | GL-1 | 105042 | Biolegend |
| CD206 | PE/CY7 | B371538 | C068C2 | 141720 | Biolegend |
| CD16 | APC/CY7 | B395722 | S17014E | 158014 | Biolegend |
| CD32B | APC | B373938 | S17012B | 156406 | Biolegend |
| CD64 | BV421 | B422389 | X54-5/7.1 | 139309 | Biolegend |
| Fiaxble viability stain | BV510 | B425259 |  | 423108 | Biolegend |

## Supplementary table 4. Antibodies for western blot

| **Markers** | **Catalogue number** | **Lot number** | **Manufacturer** |
| --- | --- | --- | --- |
| mTOR | T55306F | 333778 | Abmart |
| Phospho-mTOR | T56571F | 10011116 | Abmart |
| PI3K | T40115F | 333569 | Abmart |
| Phospho-PI3K | T55136F | 333579 | Abmart |
| NFκB | T55034 | 333579 | Abmart |
| Phospho-NFκB | 30227 | 17 | CST |
| JNK | T40073F | 10017681 | Abmart |
| Phospho-JNK | T40074F | 10009378 | Abmart |
| IκBα | T55026F | 333569 | Abmart |
| Phospho-IκBα | T562805 | 333876 | Abmart |
| Akt | T55561F | 333778 | Abmart |
| Phospho-Akt | T40067F | 333778 | Abmart |
| P38 | T55600F | 333569 | Abmart |
| Phospho-P38 | T40076F | 334471 | Abmart |
| ERK | T40071F | 333778 | Abmart |
| Phospho-ERK | T40072F | 333778 | Abmart |
| PERK | TP52759F | 10018344 | Abmart |
| Phospho-PERK | AP0886 | 5500004230 | Abclonal |
| eIF2α | A0764 | 2973020 | Abclonal |
| Phospho-eIF2α | AP0304 | 9400341001 | Abclonal |
| DDIT3 | T56694F | 10011236 | Abmart |
| Nrf2 | T55136F | 333778 | Abmart |
| Bip | T55167F | 10019082 | Abmart |
| β-Tubuin | MA8075 | 10103459 | Abmart |

**Supplementary figure 1 The purity of macrophages and procedure of cell culture.** (A) The adherent cells isolated from peripheral blood mononuclear cells (PBMC) after culture with macrophage colony-stimulating factor (MCSF) for 7 days were digested for flow cytometry. Scatter plot showed the CD68^+^ macrophages. (B) Procedure of cell culture in vitro. ITP: immune thrombocytopenia, LPS: lipopolysaccharide, IFNγ interferon γ, IL: interleukin.

**Supplementary figure 2 The flow cytometry gates of peripheral blood mononuclear cells.** Dot plots showed the gates of flow cytometry in peripheral blood mononuclear cells (PBMCs) in immune thrombocytopenia (ITP) patients and healthy controls.

**Supplementary figure 3 The GSK-3β^+^ cells and monocytes in ITP patients and healthy controls.** Dot plots showed representative flow cytometry results. (A-B) Dot plots showed the percentages of CD86^+^ M1-like monocytes (A) and CD206^+^ M2-like monocytes (B) in CD14^+^ monocytes in healthy controls and ITP patients. (C-F) The percentages of GSK-3β^+^ cells in PBMCs (C), CD14^+^ monocytes (D), CD14^+^CD86^+^ M1-like monocytes (E) and CD14^+^CD206^+^ M2-like monocytes (F) in healthy controls and ITP patients. HC: healthy controls, Pre: Newly diagnosed ITP patients, NR: ITP patients with non-response, PR: ITP patients achieving partial response, CR: ITP patients achieving complete response, CD14+/GSK3β^+^: the percentages of GSK-3β^+^ cells in CD14^+^ cells, * *P* < 0.05, ***P* < 0.01, *** *P* < 0.001.

**Supplementary figure 4 The flow cytometry gates of cultured macrophages.** Dot plots showed the gates of flow cytometry in cultured macrophages.

**Supplementary figure 5 The effects of GSK-3β inhibitor on polarization of macrophages in vitro** Macrophages derived from PBMC (peripheral blood mononuclear cells) of immune thrombocytopenia (ITP) patients were treated with different concentrations of GSK-3β inhibitor (YC-4-3). (A) The viability of M0 macrophages induced from PBMC of ITP patients, staining with Fiaxble viability stain. Contour plots showed the gates for flow cytometry. (B) Relative mRNA expression of CD163, CD80 and IL12 in macrophages treated with 10μM YC-4-3. (C-D) MFI of CD86 (C) and CD206 (D) in CD68^+^ macrophages. Histograms showed the flow cytometry plots (left). * *P* < 0.05, ***P* < 0.01, *** *P* < 0.001.

**Supplementary figure 6 The effects of GSK-3β inhibitor on the proinflammatory and phagocytosis functions of macrophages in vitro** Macrophages derived from PBMC of ITP patients were treated with different concentrations of YC-4-3. (A-C) MFI of CD16 (A), CD64 (B) and FITC-IgG-coated beads (C) under different concentrations of YC-4-3 in M0, M1 and M2 macrophages. Histograms showed the flow cytometry plots. (D-F) Scatter plots showed the gates for INFγ^+^IL-4^-^ Th1 cells and INFγ^-^IL-4^+^ Th2 cells (D), IL17+ Th17 cells (E) and CD25+Foxp3+ regulatory T cells (Tregs) (F). * *P* < 0.05, ***P* < 0.01, *** *P* < 0.001.

**Supplementary figure 7 The original figures of western blot** Figure A-E derived from the same member, and figure F-J derived from the same member. (A) Figure showed the protein markers (EpiZyme, Shanghai, China, Cat: WJ102) and all the protein lanes in manuscript (red arrow). (B) Original protein lanes of P-mTOR, P-PERK and Bip, exposure time was 9 second. (C) Original protein lanes of P-NFκB, and P-P38, exposure time was 1 second. (D) The member in A was stripped and re-incubated with anti-β-Tubulin and DDIT3. Figure showed the protein markers (EpiZyme, Shanghai, China, Cat: WJ102) and all the protein lanes in manuscript (red arrow). (E) Original protein lanes of β-Tubulin and DDIT3, exposure time was 1 second. (F) Figure showed the protein markers (EpiZyme, Shanghai, China, Cat: WJ102) and all the protein lanes in manuscript (red arrow). (G) Original protein lanes of mTOR, and PERK, exposure time was 11 second. (H) Original protein lanes of NFκB, and P38, exposure time was 8 second. (I) The member in F was stripped and re-incubated with anti-β-Tubulin. Figure showed the protein markers (EpiZyme, Shanghai, China, Cat: WJ102) and all the protein lanes in manuscript (red arrow). (J) Original protein lanes of β-Tubulin, exposure time was 5 second.

#
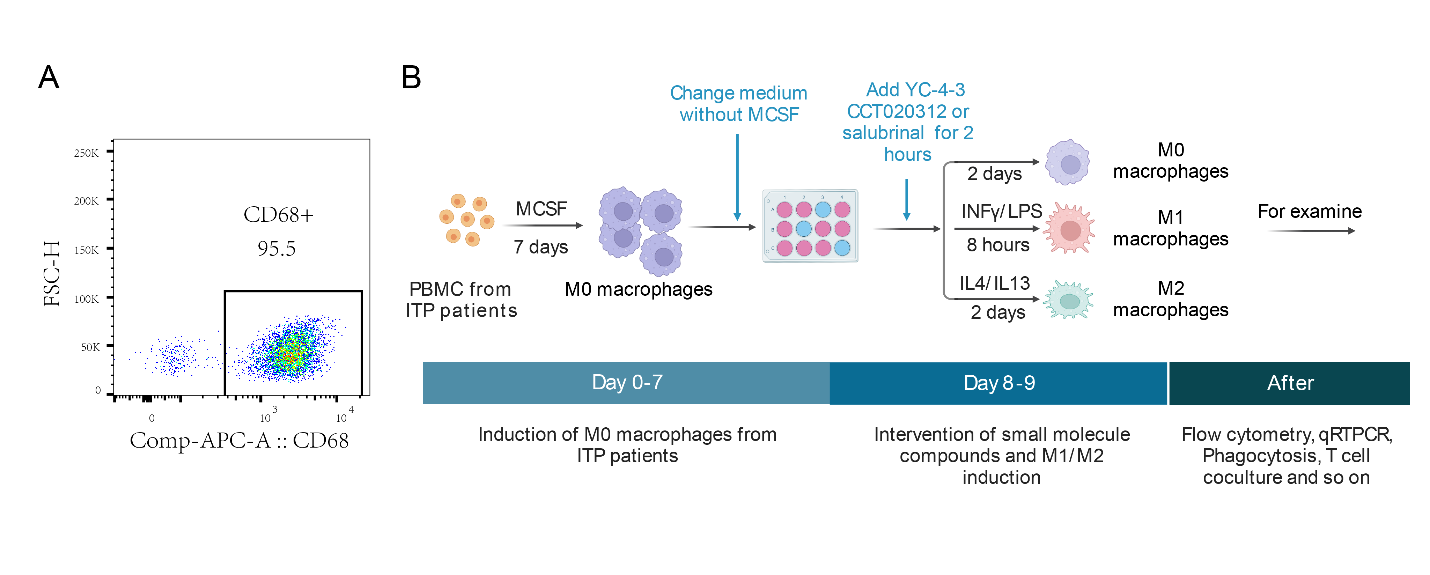
Supplementary figure 1

# Supplementary figure 2


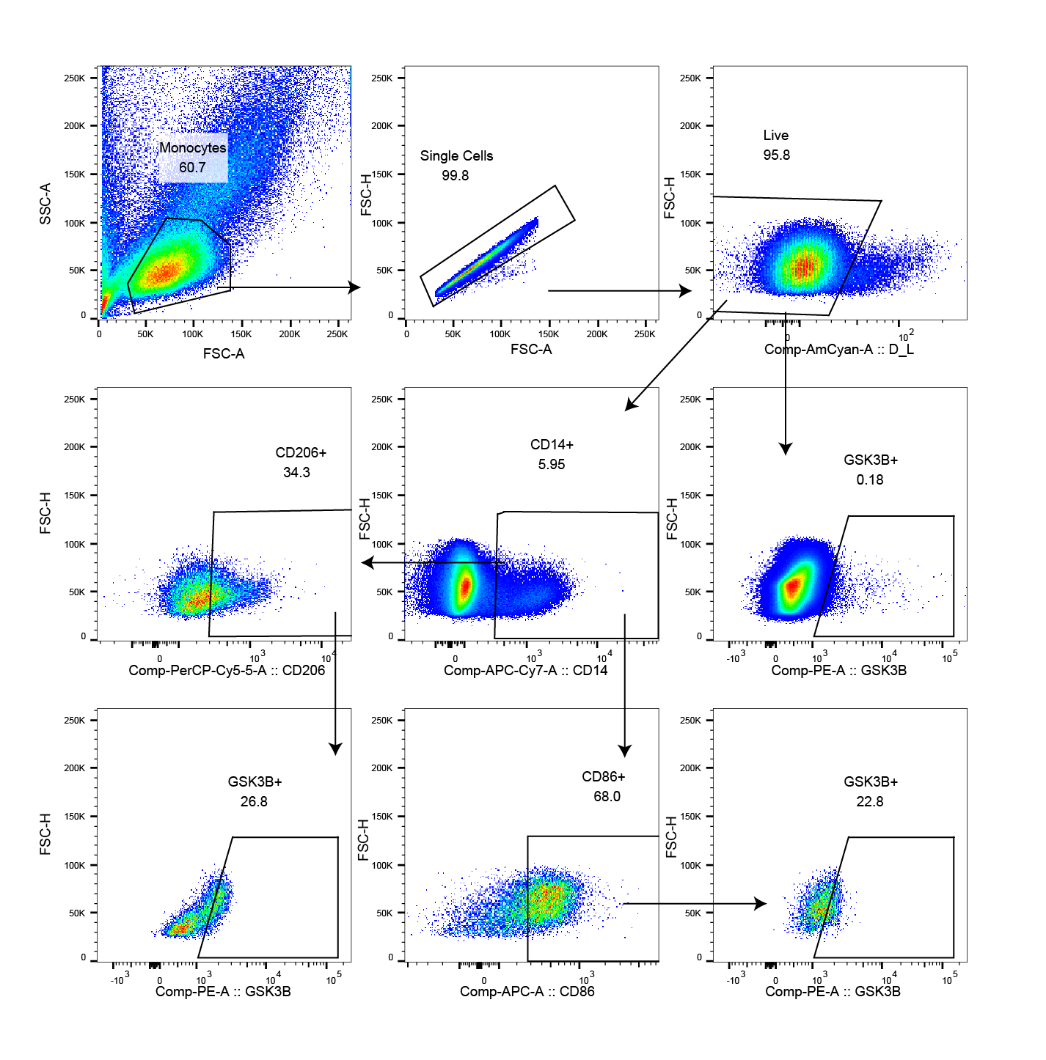


# Supplementary figure 3


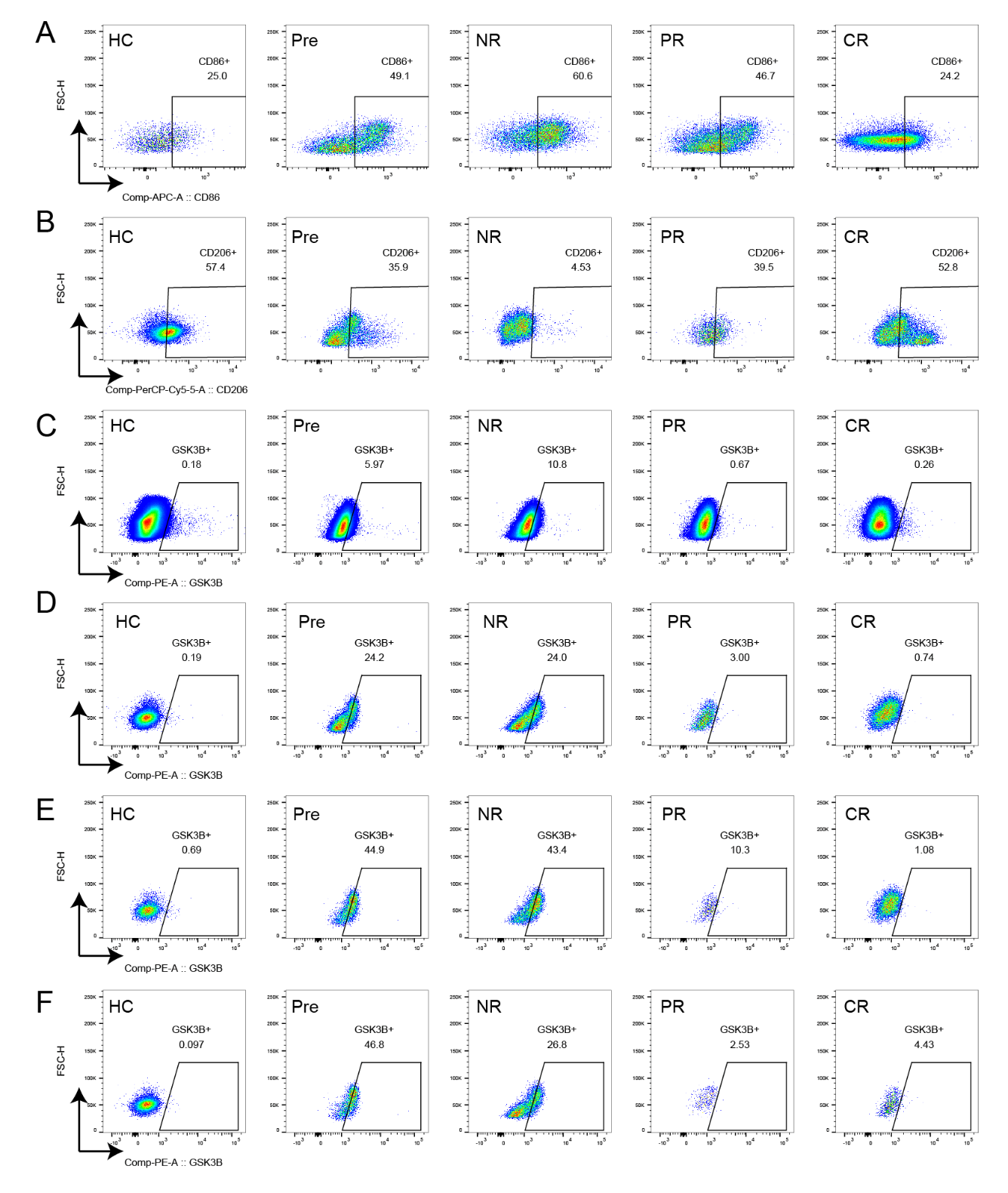


# Supplementary figure 4


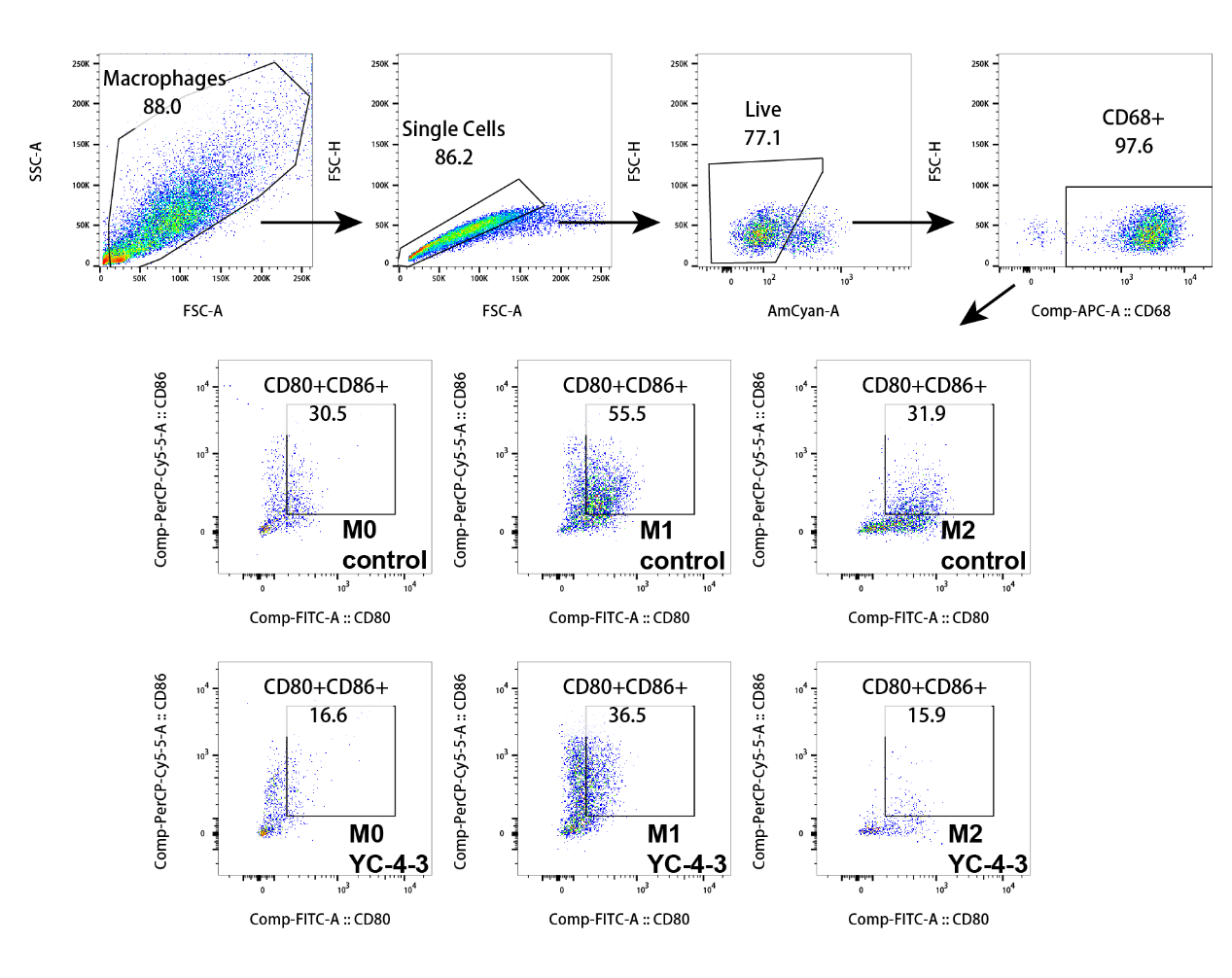


#
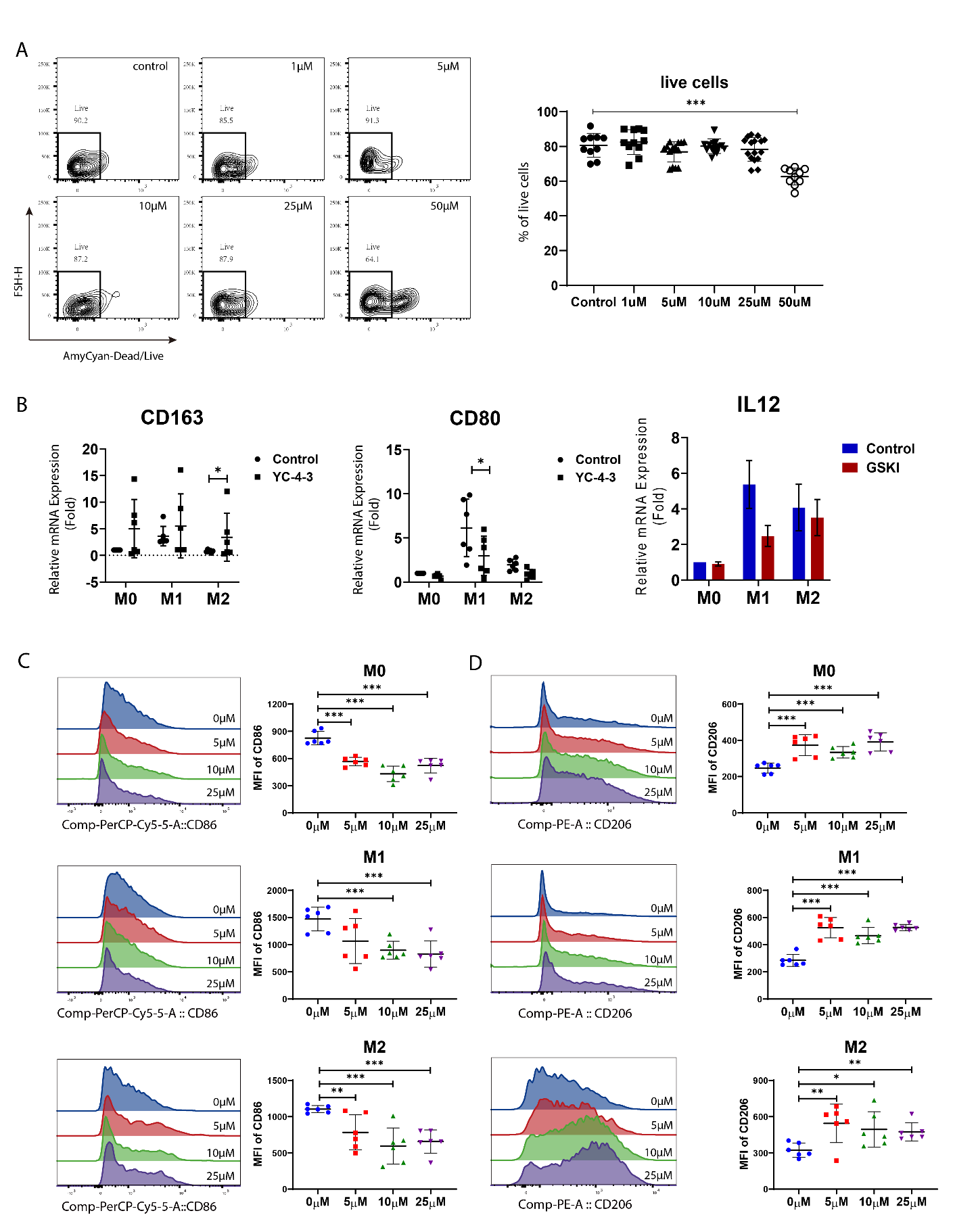
Supplementary figure 5

#
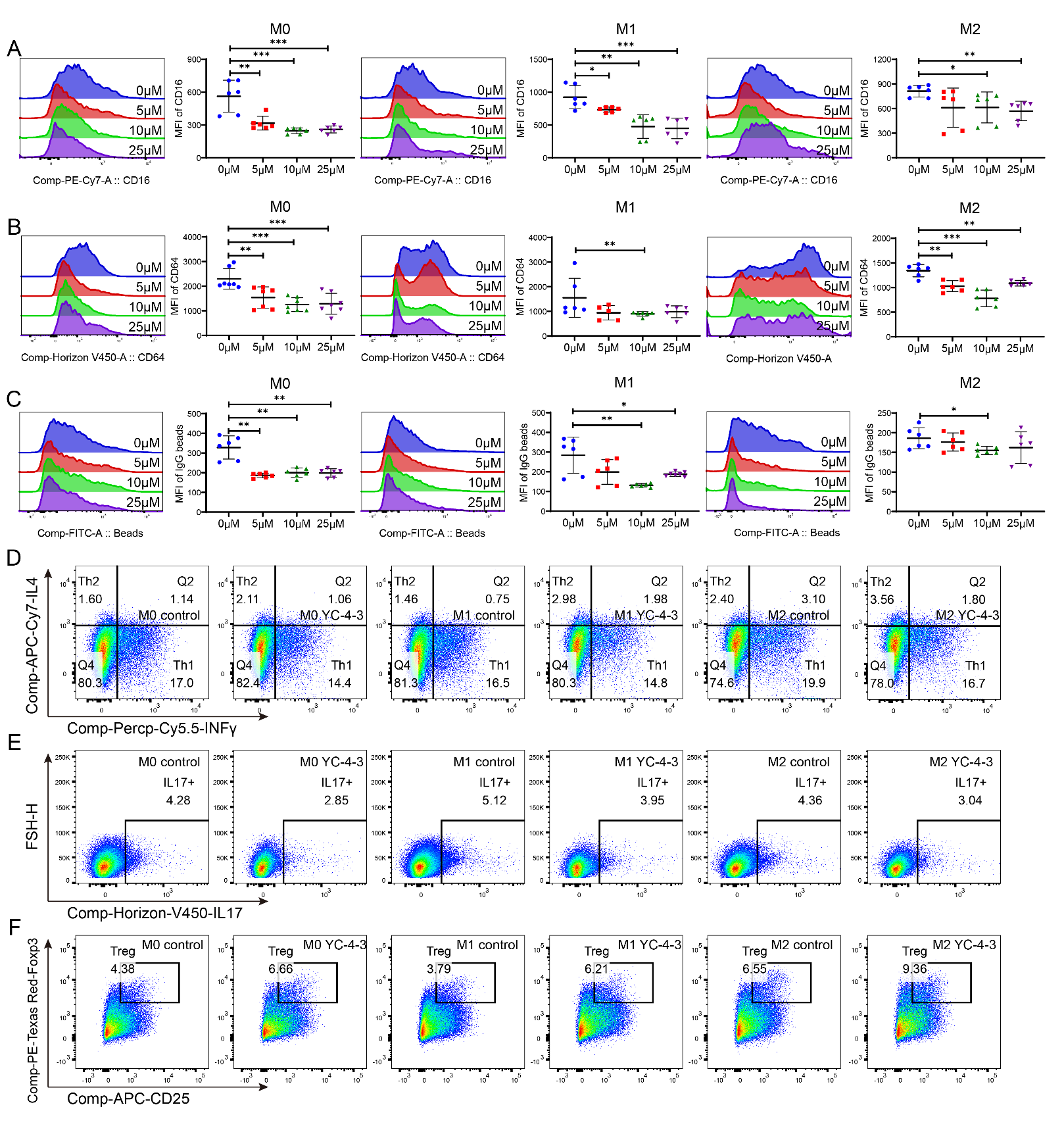
Supplementary figure 6

# Supplementary figure 7


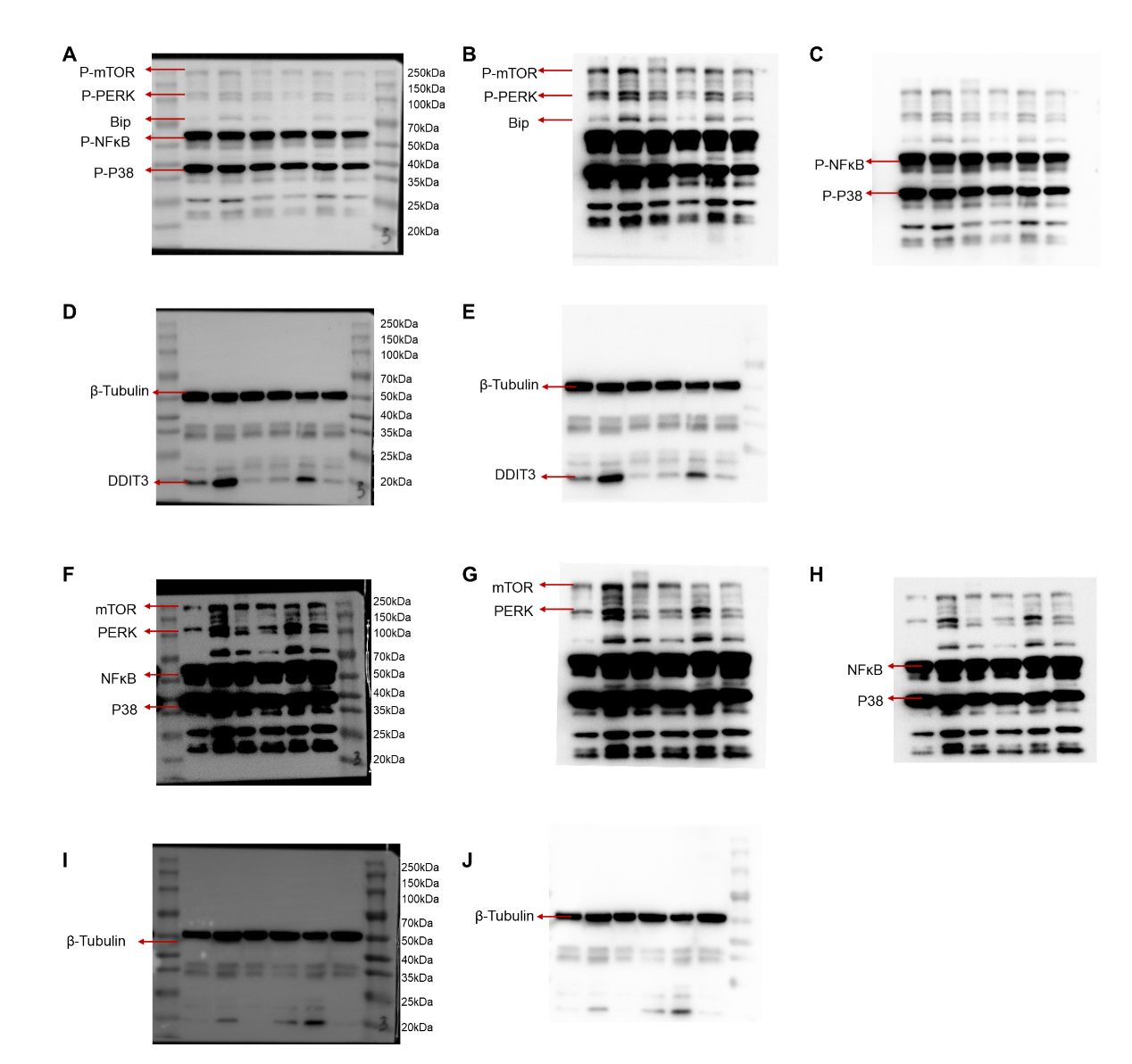

Supplement: Supplementary file 1 — Supporting Information [file ADVS-12-2412515-s001.docx]
